# Supplementary material for: Surface Properties of 1DTiO2 Microrods Modified with Copper (Cu) and Nanocavities
Source: Nanomaterials (Basel). 2021 Jan 27;11(2):324. doi: 10.3390/nano11020324 (PMC7912526; doi:10.3390/nano11020324)
Supplement: Supplementary file 1 [file nanomaterials-11-00324-s001.pdf]

<u>Supplementary Material</u>

Surface properties of 1D TiO_2 microrods modified with copper (Cu) and nanocavities

1. Thermal analysis and mass spectra

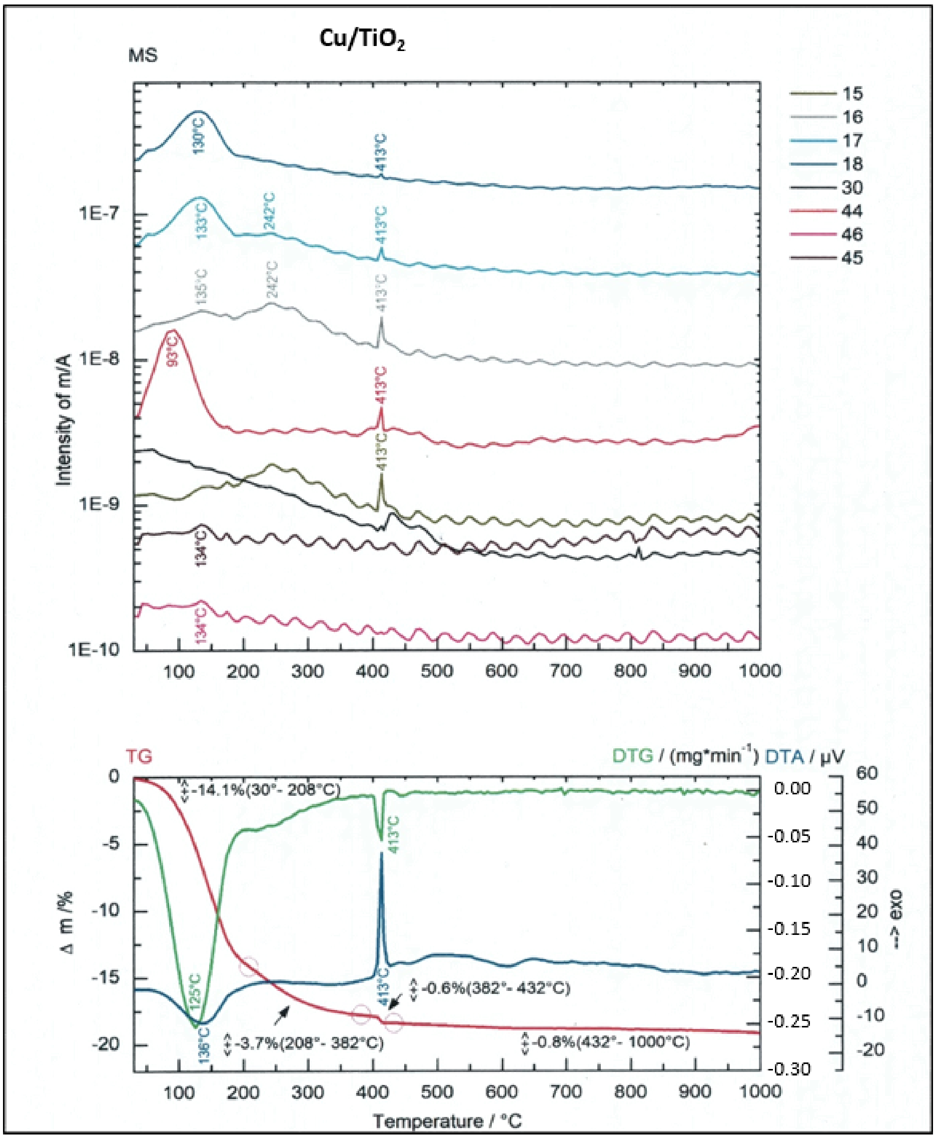

Figure S1 Results of thermal and MS analysis

Figure S1 captures the results of the thermal analysis of a Cu /TiO_2_N sample (simultaneous TG/DTA curves). The DTA curve shows the minimum at 136 ° C belonging to the endothermic reaction (water release) and the maximum at 413 ° C belonging to the exothermic reaction (adsorbed OH groups on the surface). It can be

seen from the TG curve that the weight loss after heating is approximately 19%. This is due to the release of gaseous species of H_2O with evolution curves 18, 17, 16**.** In the second and third steps, the escape of CO_2 (evolution curves 44,16,45,46), NH_3 (evolution curves 16,17,15), N_2O (evolution curves 44, 30, 16), NO (15), and SO_2/SO (evolution curves 48/64) from the Cu /TiO_2_N sample were observed [1]. The sample mass spectra (MS) with lines indicated the peaks of each species by using the NIST Mass Spectral Search Program (Software version 2.2) [2] are presented in Figures S2-S10.

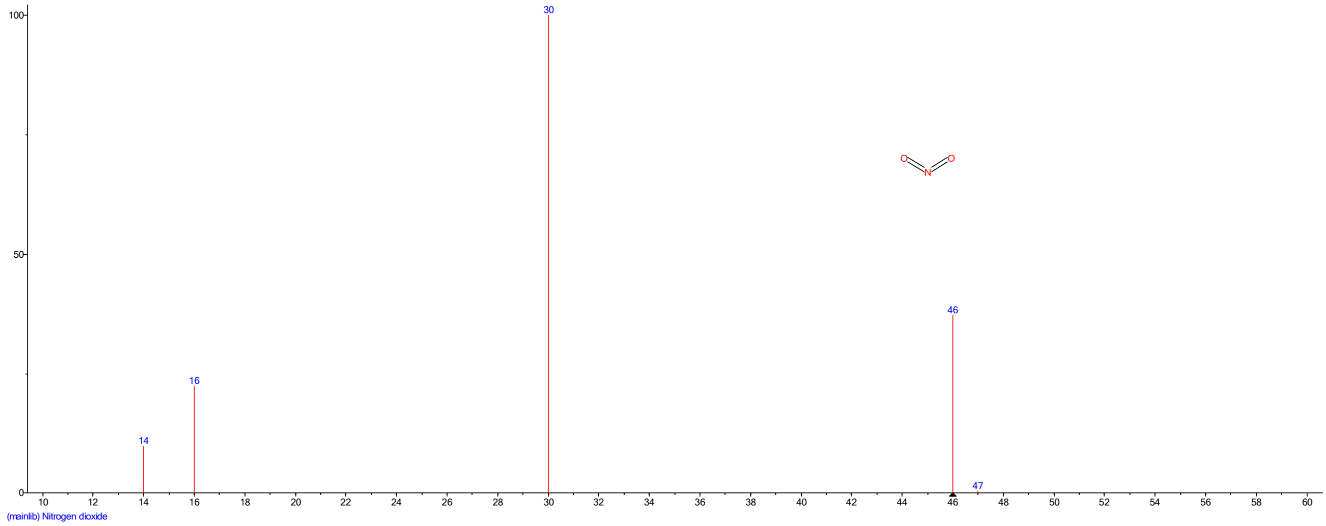

Figure S2. Evolution of gas fragments of NO_2

5 largest peaks:

30 999 | 46 370 | 16 223 | 14 96 | 47 1 |

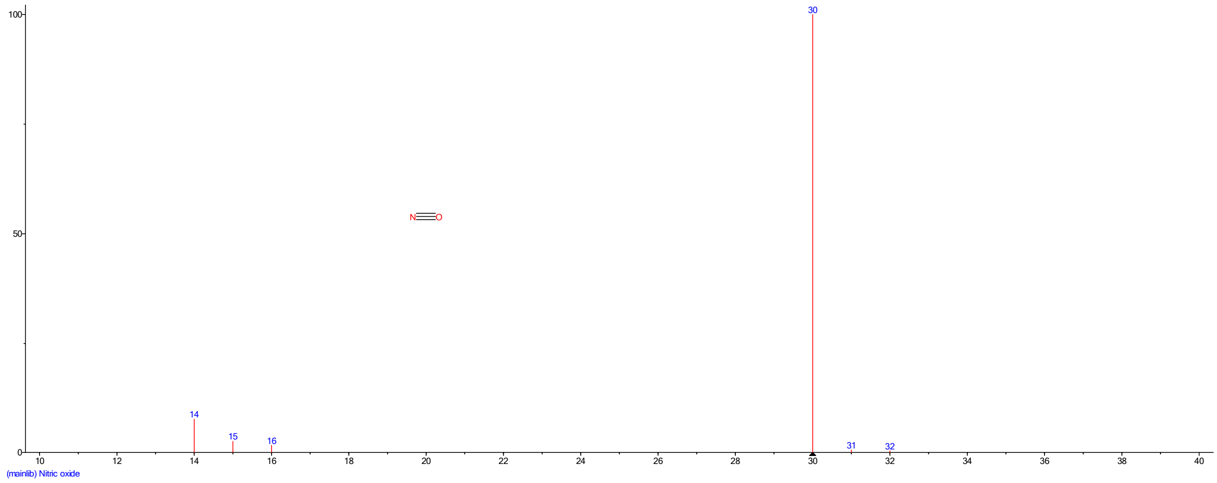

Figure S3. Evolution of gas fragments of NO

6 largest peaks:

30 999 | 14 75 | 15 24 | 16 15 | 31 4 | 32 2 |

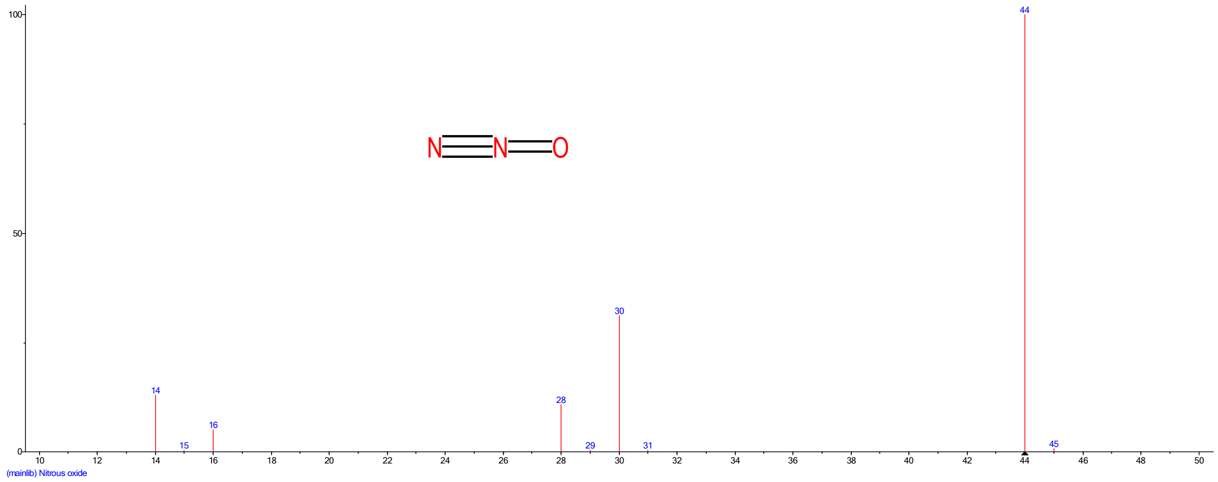

Figure S4. Evolution of gas fragments of N_2O

9 largest peaks:

44 999 | 30 311 | 14 129 | 28 108 | 16 50 |
45 7 | 15 1 | 29 1 | 31 1 |

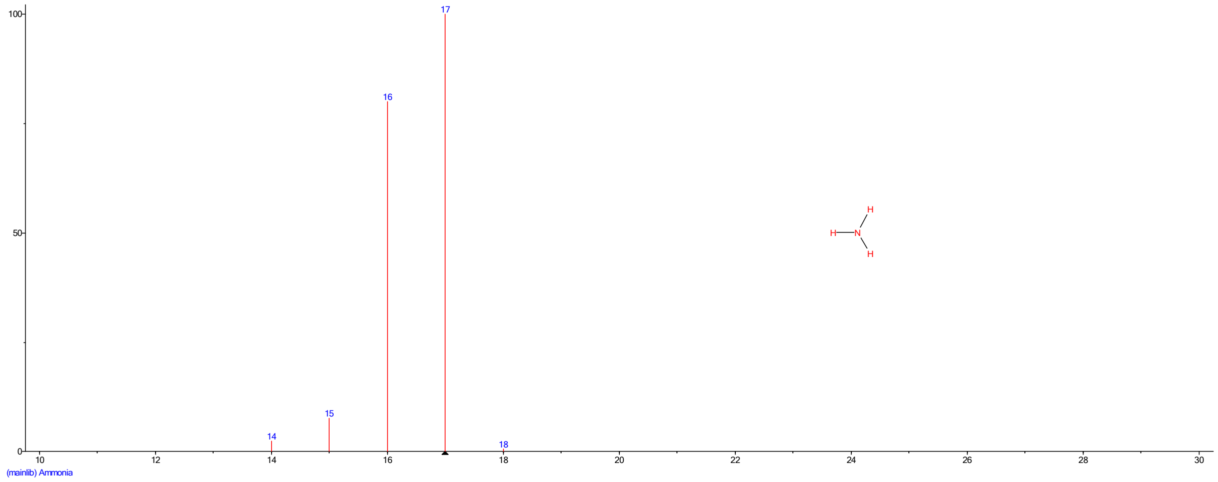

Figure S5. Evolution of gas fragments of NH_3

5 largest peaks:

17 999 | 16 800 | 15 75 | 14 22 | 18 4 |

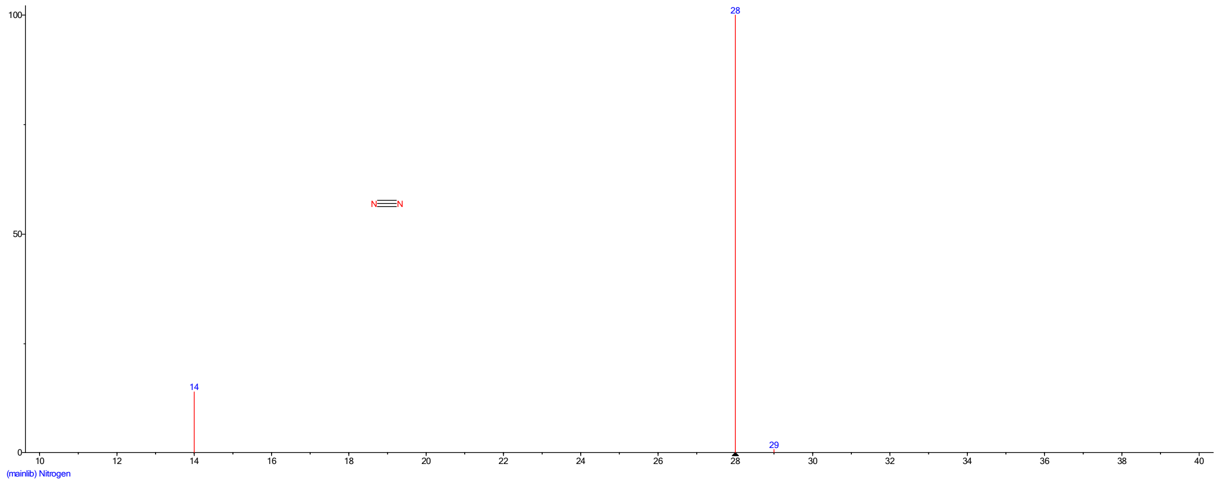

Figure S6. Evolution of gas fragments of N_2

3 largest peaks:

28 999 | 14 137 | 29 7 |

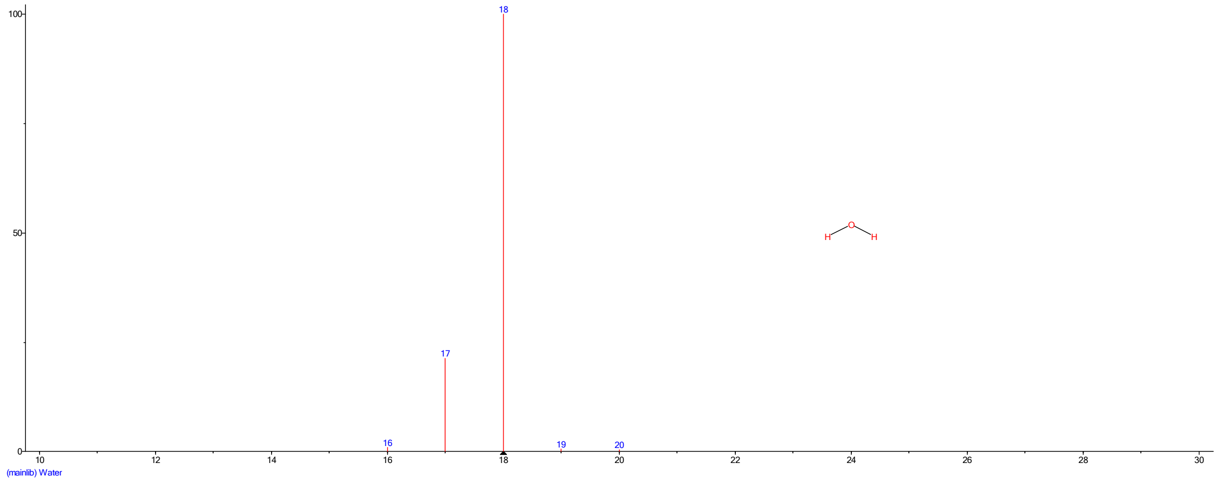

Figure S7. Evolution of gas fragments of H_2O

5 largest peaks:

18 999 | 17 212 | 16 9 | 19 5 | 20 3 |

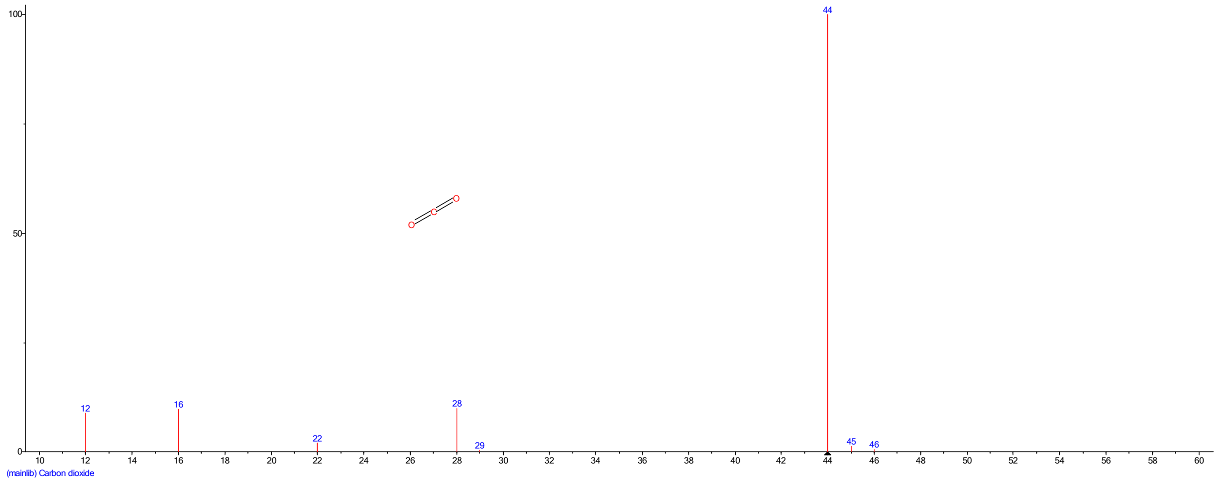

Figure S8. Evolution of gas fragments of CO_2

8 largest peaks:

44 999 | 28 98 | 16 96 | 12 87 | 22 19 |
45 12 | 46 4 | 29 1 |

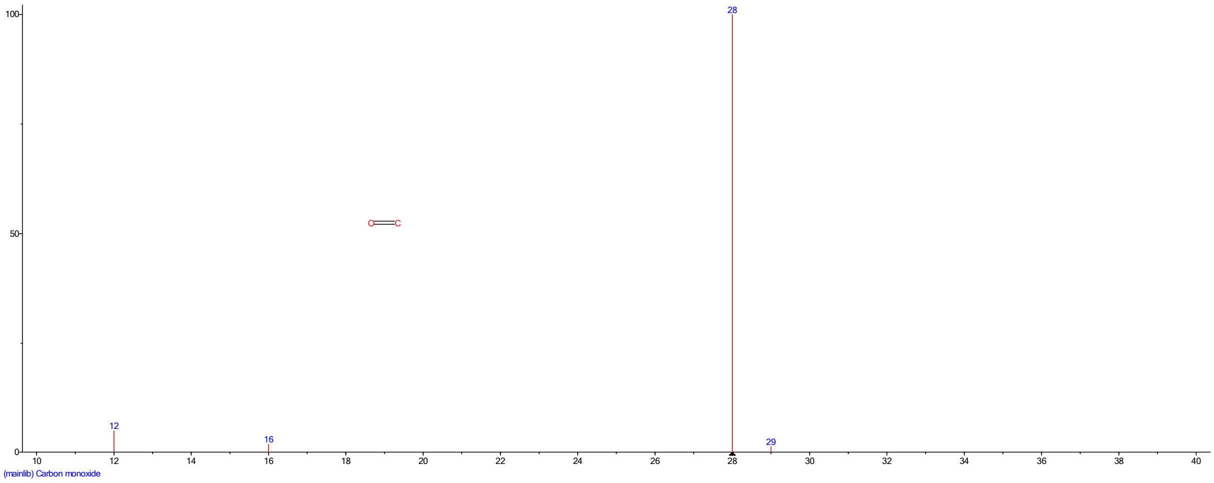

Figure S9. Evolution of gas fragments of CO

4 largest peaks:

28 999 | 12 47 | 16 17 | 29 12 |

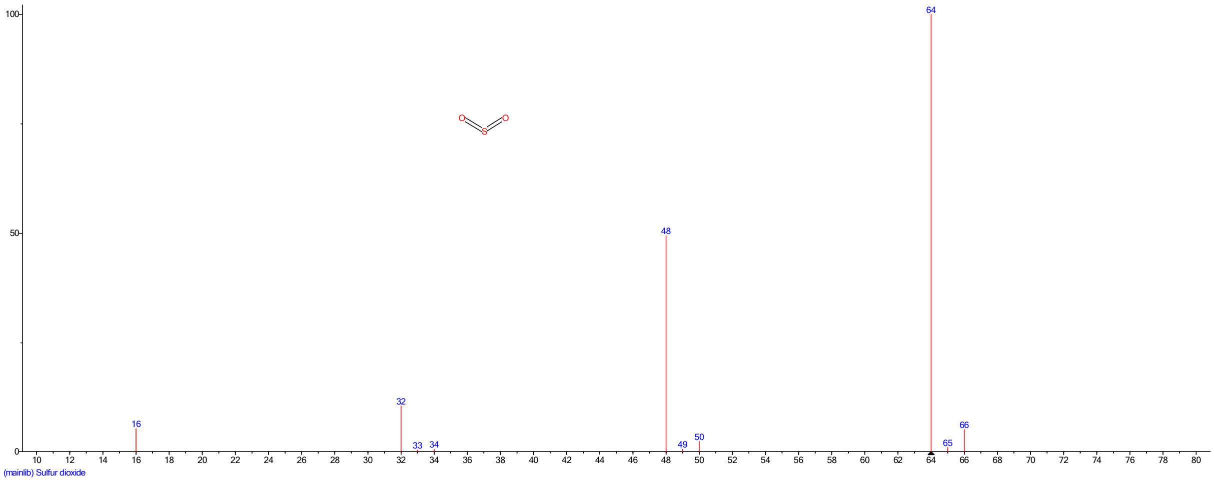

Figure S10. Evolution of gas fragments of SO_2

10 largest peaks:

64 999 | 48 493 | 32 104 | 16 52 | 66 49 |
50 23 | 65 9 | 34 4 | 49 4 | 33 1 |

REFERENCES

[1] Madarász, J., Varga, P., Pokol, G., Evolved gas analyses (TG/DTA–MS and TG–FTIR) on dehydration and pyrolysis of magnesium nitrate hexahydrate in air and nitrogen, J Anal and Applied Pyrolysis, 2007, 79, 475–478.

[2] Stein, S., Mrokhin, Y., Tchekhovkoi, D., Mallard, W., NIST/EPA/NIH Mass Spectral Library with NIST Mass Spectral Search Program (Software version 2.2) 2014, NIST, the U.S. Secretary of Commerce.
